# Ultrastructural analysis of throat dermal tissue and chromatophore components in the threespine stickleback (*Gasterosteus aculeatus*)

**DOI:** 10.7717/peerj.16248

**Published:** 2023-12-05

**Authors:** Christopher M. Anderson, Thomas Fink, Jeffrey S. McKinnon

**Affiliations:** Department of Biology, East Carolina University, Greenville, NC, United States of America

**Keywords:** Threespine stickleback, *Gasterosteus aculeatus*, Dermal chromatophore, Animal coloration, Histology, Ultrastructure

## Abstract

The threespine stickleback (*Gasterosteus aculeatus*) is an important model for studying the evolution of nuptial coloration, but histological analyses of color are largely lacking. Previous analyses of one nuptial coloration trait, orange-red coloration along the body, have indicated carotenoids are the main pigment producing this color. In addition, recent gene expression studies found variation in the correlates of throat coloration between the sexes and between populations, raising the possibility of variation in the mechanisms underlying superficially similar coloration. We used transmission electron microscopy (TEM) to investigate the histological correlates of color in the throat dermal tissue of threespine stickleback from Western North America, within and between sexes, populations, and ecotypes. Ultrastructural analysis revealed carotenoid-containing erythrophores to be the main chromatophore component associated with orange-red coloration in both males and females across populations. In individuals where some darkening of the throat tissue was present, with no obvious orange-red coloration, erythrophores were not detected. Melanophore presence was more population-specific in expression, including being the only chromatophore component detected in a population of darker fish. We found no dermal chromatophore units within colorless throat tissue. This work confirms the importance of carotenoids and the erythrophore in producing orange-red coloration across sexes, as well as melanin within the melanophore in producing darkened coloration, but does not reveal broad histological differences among populations with similar coloration.

## Introduction

The study of color pattern evolution (reviewed in Endler & Mappes, 2017) has been transformed in recent decades by advances in the quantification of coloration and improvements in methods for evaluating how colors are perceived by animals with visual pigments substantially different from our own (*e.g.*, Endler & Mielke Jr, 2005; Håstad, Victorsson & Ödeen, 2005; Champ, Vorobyev & Marshall, 2016; Rennison et al., 2016; Maia et al., 2019; Van den Berg et al., 2020). These sophisticated analyses of the appearance of color patches and patterns are being complemented by investigations of the genetic and/or histological basis of coloration (*e.g.*, Fujii, 1993; Hoekstra, 2006; Greenwood, Cech & Peichel, 2012; Kottler et al., 2014; Yong, Peichel & McKinnon, 2016; Hart & Miller, 2017). Together, these approaches are enabling more robust inferences about color evolution at the level of genes and development (*e.g.*, Daly et al., 2013; Mallarino et al., 2016; Parichy, 2021).

Within poikilothermic vertebrates, the basic unit of coloration is the dermal chromatophore (Fujii, 1993). This structural unit is comprised of three layers, which include (i) the superior most erythrophore cells (a term we will henceforth use in an inclusive way to refer to structures classically termed either ‘xanthophore’ or ‘erythrophore’) containing pteridine and/or carotenoid pigments; (ii) iridophore cells containing light-reflecting crystalline platelets just beneath; and (iii) melanophore cells containing melanin pigments, lying deepest in the tissue (Bagnara, Taylor & Hadley, 1968; Taylor & Bagnara, 1972; Bagnara & Hadley, 1973). Changes in the quantity and/or arrangement of pigment types and chromatophore components can result in altered coloration (reviewed in Grether, Kolluru & Nersissian, 2004*)*, making study of the dermal tissue, chromatophore presence, and pigmentation within the chromatophore essential when addressing how color production/variation evolves.

The threespine stickleback (*Gasterosteus aculeatus)* has long been an important model for evolutionary and molecular studies of coloration (*e.g.*, Wedekind et al., 1998; Greenwood et al., 2011; Malek et al., 2012; Yong, Peichel & McKinnon, 2016; McKinnon, Kitano & Aubin-Horth, 2019), but studies of the histological basis of stickleback color variation have been limited (though see Burton, 1978; Burton, 1979). This species complex has undergone repeated adaptive radiations that have resulted in phenotypically diverse populations distributed across the northern hemisphere (Bell & Foster, 1994; McKinnon & Rundle, 2002). Males often exhibit striking nuptial coloration during the breeding months, typically including iridescent blue eyes, green-blue dorsolateral coloration, and orange-red coloration along the ventral and lateral portions of the body and throat (McLennan & McPhail, 1990; Bakker & Milinski, 1993; Bell & Foster, 1994; Rush et al., 2003; Flamarique et al., 2013; Candolin & Tukiainen, 2015). Despite nuptial coloration being largely sexually dimorphic in expression, there is evidence of orange-red throat and/or spine coloration in both sexes in multiple localities (*e.g.*, McKinnon et al., 2000; Amundsen et al., 2015; Yong, Peichel & McKinnon, 2016; Kroken et al., 2021; Anderson & McKinnon, 2022).

Orange-red nuptial coloration in sticklebacks is thought to be primarily the result of carotenoids, with the type and amount of carotenoids present causing variation from weak orange to red (Brush & Reisman, 1965; Wedekind et al., 1998). Many investigations have addressed the evolution and function of orange-red throat and lateral coloration in threespine stickleback (Rowland, 1982; Bakker & Mundwiler, 1994; McKinnon, 1996). Other studies have characterized the carotenoids involved in color production (Wedekind et al., 1998; Nordeide, Rudolfsen & Egeland, 2006; Pike et al., 2011) and the genetic underpinnings of pigmentation (Malek et al., 2012; Yong, Peichel & McKinnon, 2016). However, no work has yet characterized histological features of stickleback throat dermal tissue (though see Burton, 1975; Burton, 1978; Burton, 1979 for histological investigations into skin structure and lateral body melanophore analysis) with regard to coloration.

Such work is called for by recent RNAseq results, which suggest differentiation in the gene expression correlates of throat color (McKinnon, Newsome & Balakrishnan, 2022). Researchers identified gene expression correlates of female red intensity within populations, but the correlated genes differed between populations (McKinnon, Newsome & Balakrishnan, 2022). Interpopulation comparisons detected candidate genes previously found to be involved in carotenoid pathways, such as TTC39B, to be significantly associated with orange-red coloration for both sexes, but sex and population specific genes were also identified (McKinnon, Newsome & Balakrishnan, 2022). These results raise the question of whether similar stickleback color patterns may result from, or be mediated by, different genetic and possibly cellular mechanisms.

Beyond pigmentation, it has also been shown that collagen fibrils, which are often abundant in human and animal dermal tissue (*e.g.*, Fox & Whitear, 1990; Matsuda et al., 2006), can alter reflectivity in skin (Brightman et al., 2000). Collagen fibrils could potentially contribute to stickleback throat coloration.

The primary goal of this investigation was to characterize the chromatophore components, pigment cell distribution, and collagen fibril presence within *G. aculeatus* throat dermal tissue through the use of transmission electron microscopy. We sought to compare these characteristics across color variants, as well as sexes, populations, and life histories (*i.e.,* anadromous *vs* freshwater). We asked, in particular, whether similar or distinct histological features are associated with similar coloration, including between the sexes and in independently evolving populations. Based on studies to date, variation in carotenoid-containing erythrophores is expected to mainly account for production of orange-red color within both sexes and all populations containing any red coloration. We also investigated the potential roles of other cell types, particularly melanophores, which should be especially important in fish with dark coloration but could also play a role in the production of red coloration (Miller et al., 2007; Greenwood et al., 2011; Greenwood, Cech & Peichel, 2012; Hart & Miller, 2017), as well as collagen fibrils.

### Materials & Methods

### Stickleback collection and maintenance

Using minnow traps, adult male and female threespine stickleback were collected from four populations in British Columbia, which were selected to encompass diversity in ventral throat color (Table 1), including three mainland sites: (i) a downstream (anadromous) site of the Little Campbell River (49.016N, 122.778W; May 2018); (ii) an upstream (freshwater) site of the Little Campbell River (49.012N, 122.625W; May 2018); (iii) Salmon River (49.091N, 122.493W; May 2017 and 2018); as well as one site on Vancouver Island, (iv) Bonsall Creek drainage (48.851N, 123.712W; April 2014). All fish were transported live to our lab at East Carolina University (Greenville, NC, USA) and allowed to acclimate for >3 weeks.

**Table 1:** Throat color phenotype across populations and sexes. Populations and collection sites were chosen based upon the intensity of orange-red throat coloration. Little Campbell Anadromous is known to contain males with highly red-colored throats and females lacking color. Both Little Campbell Stream and Salmon River freshwater sites are known to contain both males and females with red-colored throats, as well as dull females, though Salmon River tends to have fewer, less colorful females. Bonsall Creek is known to contain dull colored males and females, in the sense of generally lacking orange-red coloration, but breeding males appear to be darker in appearance (*i.e.* melanic).

**Collection site**	**Males**			**Females**		
	**Dull**	**Colorful**	**Melanic**	**Dull**	**Colorful**	**Melanic**
Little Campbell Anadromous	X	X				
Little Campbell Stream		X		X	X	
Salmon River		X		X	X	
Bonsall Creek			X	X		

Fish were housed within our aquatic facility at 17–20 °C in single population, mixed-sex 99-L tanks (92 cm × 33 cm × 33 cm) that contained limestone aquarium rocks, an air stone, and a single sponge filter at approximately 15–20 fish per tank. Natural-spectrum mimicking fluorescent lights (Lumicrhome^®^ Full Spectrum Plus, Lumiram Electric Co, Larchmont, NY, USA) were used to illuminate tanks for a 15-hour photoperiod at the time of analysis. Fish were fed twice daily with a combination of bloodworms (chironomid larvae) and brine shrimp. Only healthy fish, as evidenced by normal behavior and the absence of any sign of infection, were utilized for analysis.

All photography and histological processing occurred during the summer months of 2018. Collection approval was granted by the British Columbia Ministry of Forests, Lands, and Natural Resource Operations (permits NASU14-92909, MRNA17-262956, MRSU18-290075). This research was approved and followed the guidelines governed by the Institutional Animal Care and Use Committee (IACUC) of East Carolina University (Animal Use Protocols #D224 and #D349).

### Color assessment—photography and visual based scores

Color of dermal throat tissue was assessed using a visual scale. Immediately upon removal from the water, fish were gently dabbed to remove excess water and placed into a custom sponge that acted to hold the body of the stickleback still while exposing the ventral surface of the throat. Photographs of the ventral throat were taken using a digital camera (Cannon DS126171) mounted on a copy stand directly above the live fish. Each fish was illuminated with Solux Halogen lamps (Tailor Lighting Inc., Rochester, NY, USA) on either side of the camera and angled at 45 degrees. All fish were photographed against an 18% grey card for standardization.

**Figure 1:**
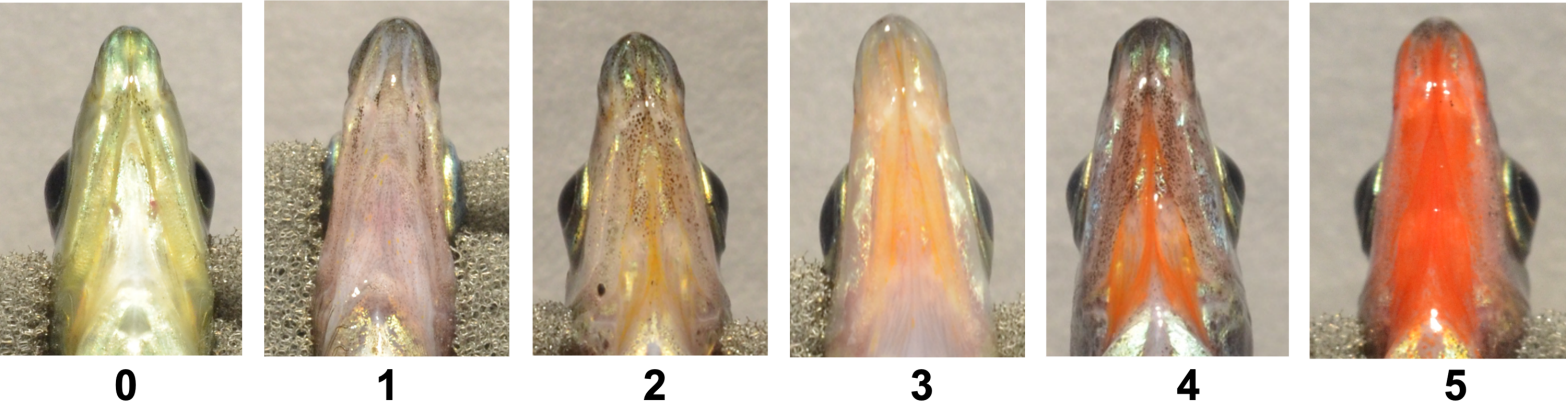
Visual based throat color scores. Fish were characterized based on a visual based system, with color rankings: 0 (dull, colorless), 1 (dull color with no macroscopic orange-red expression), and 2–5 (colorful range of broad orange-red expression). Individuals pictured represent various sexes, color morphs, populations, and ecotypes.

All fish were visually scored on a scale of 0-5 by a single researcher (CMA) immediately upon being photographed. Scores were broken into two main categories, *i.e.,* “dull” and “orange-red”. Scores of 0 and 1 indicated dull coloration, with 0 describing colorless tissue and 1 describing slightly darkened tissue void of orange-red coloration. Scores of 2–5 indicated the presence of orange-red coloration with increasing intensity (Fig. 1). Any obvious black coloration was also noted.

### Biological tissue processing for transmission electron microscopy

Two fish from each sex, population, and color expression type (*i.e.,* colorful or dull) found within a population were randomly selected for further inclusion in the study and biological tissue processing, *i.e., n* = 8 males and *n* = 12 females. Sample preparation methods for biological tissue processing were largely based on Mascorro & Bozzola (2007). Stickleback were removed from their tanks, photographed, then immediately placed into a lethal dose of MS-222 for 7 min, with no evidence of color reduction occurring during this time. The spinal cords of all fish were then cut and the heads were removed and placed into a 3.125% glutaraldehyde fixative solution with 0.1 M sodium phosphate (Sorenson’s buffer; ph 7.2) at 5–10 °C for 1–2 h. Once this time elapsed, the heads were removed from solution, and the ventral throat tissue was excised and placed back into the primary fixative before washing.

Tissues were then washed thoroughly in 0.1 M Sorenson’s buffer. This process was repeated three times for a period of 15 min with solution changes between each. Next, tissues went into post-fixation solutions of 1% Osmium tetroxide in 0.1 M Sorenson’s buffer for 1–2 h before being rinsed three times with deionized water for 1–3 min each round. Tissue samples then went through a dehydration series to remove all water from the tissue, which is a necessary step for infiltration. This series included washes of 50%, 75%, 95%, and 100% ethanol solutions for 15 min each, followed by two final rinses of 100% ethanol at 10 min each.

Once dehydrated, tissue samples were placed into a room temperature 1:1 SPURRS/Epoxy solution for 1 h, then transferred into a 100% SPURRS solution for 3–15 h for infiltration prior to embedding. All tissue processing, from primary fixative through infiltration, occurred on gyratory rockers to ensure movement of solution and effective processing of the tissue itself.

**Figure 2:**
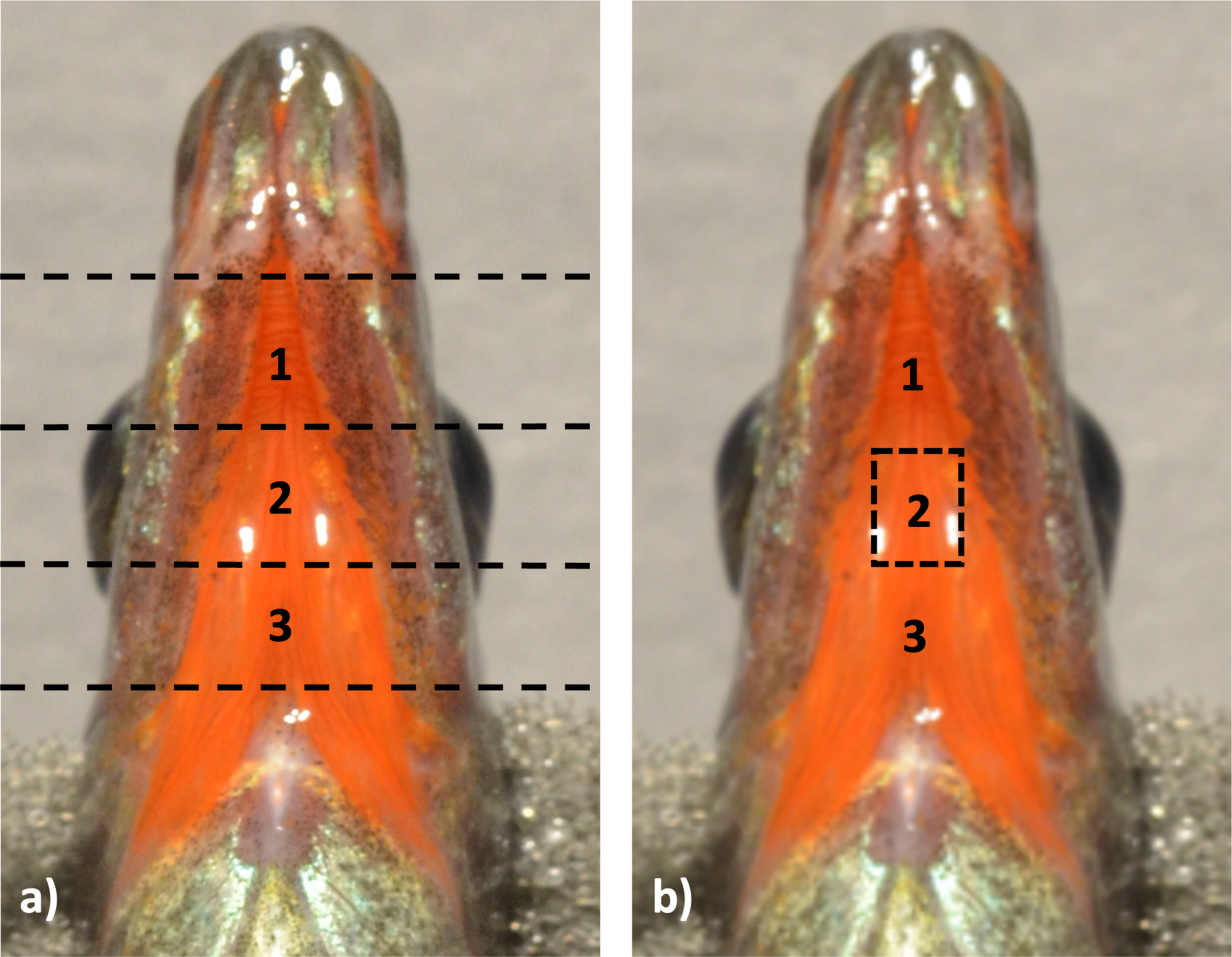
Throat tissue transection for TEM analysis. Throat tissue was dissected off the fish at the start of the biological processing stages, and then (A) differentiated into three portions using horizontal transections, dividing the throat into anterior, posterior, and middle sections. The middle most section (B) was then used in this work for further analysis of dermal structure.

Next, throat tissues were placed ventral side up and transected into three sections along the anterior-posterior axis (Fig. 2), leaving tissue sections ∼3 mm × 3 mm in size. The most anterior and posterior portions of the tissues were fixed separately from the middle portion of the throat, which was the area of most interest for this study and the only section analyzed (Fig. 2B).

All sections of the throat were transferred into silicone-rubber flat block embedding molds containing 100% SPURRS, with the ventral side facing upwards, and allowed to polymerize for 15 h in a 65 °C oven. Once samples polymerized completely, as evidenced by the hardness of the material, the blocks were removed, individually marked with an ID, and stored at room temperature.

Blocks from the middle section of the throats were trimmed to 1 mm × 1 mm in size by removing 1 mm of tissue from all sides of the 3 mm × 3 mm transected tissue, leaving only the middle most tissue. Using a Leica ultramicrotome, thick sections (∼1 µm) were collected and analyzed using light microscopy to ensure dermal presence. Ultra-thin sections (∼90 nm) were then collected, with 6–8 sections per grid, and 3–4 grids per fish. Grids containing these sections were then stained using uranyl acetate and lead citrate washes for optimal imaging under the TEM.

### Transmission electron microscopy

Transmission electron microscopy (TEM) was conducted from Fall 2018–Spring 2020. Prepared tissue grids were randomly selected from each individual (*n* = 20) for inclusion in TEM. At the time of microscopy, both microscopists (CMA and TF) were blind to the color score of the fish. Images were taken at standard magnifications between 4,000x–45,000x. One grid containing multiple concurrent sections was analyzed per individual.

Upon completion of microscopy, one sample was removed from further analysis due to flaws in all micrographs that would not allow clear identification of structures within, leaving *n* = 19 total individuals analyzed further.

### Identification of dermal chromatophore units

Dermal chromatophore units are often characterized by topographic and/or structural components (see Bagnara, Taylor & Hadley, 1968; Taylor & Hadley, 1970; Grether, Kolluru & Nersissian, 2004 and citations within). The topographic identification leads to characterization of the layers based upon their locations and the orientation of the chromatophore units and placement within the tissue section, but requires multiple chromatophore layers to be in close proximity to each other. Beyond this topographic system of chromatophore layer identification, they can be defined by the characteristics of the structures contained within the chromatophore components.

Because tissue from fish in this study did not reveal any classical chromatophore units, topographic identification was not possible. Instead, the following definitions were used to identify pigment-containing chromatophore components. First, a chromatophore was determined to be a melanophore if (i) all structures within were sized up to 500 nm, circular or ovular in shape, and black in appearance, or (ii) if the structures within varied in color from light to black, then any non-black structure should present either stripes or black granules clustered within the border of the circular structure as characterized by the different stages of melanogenesis (*e.g.*, Hirobe & Abe, 2007; Raposo & Marks, 2007; Xiao, Zhang & Zhu, 2019). A chromatophore was characterized as an erythrophore if the above conditions were not met; that is if (i) the layer contained circular structures that were not all starkly black, but rather varied in color from near colorless (electron light) to dark (electron dense) and (ii) the circular structures did not present striped patterns or contain notable black granules evident of the different stages of melanogenesis. While the erythrophore layer is classically characterized by clear-milky appearing droplets, some studies have also found darker structures, which appeared close to what we detected in stickleback throat dermal tissue, within this characterized layer (Phang, Lim & Khoo, 2012; Paray & Al-Sadoon, 2017; Sušnik Bajec et al., 2022) and helped us to form the basis for these applied definitions.

## Results

**Figure 3:**
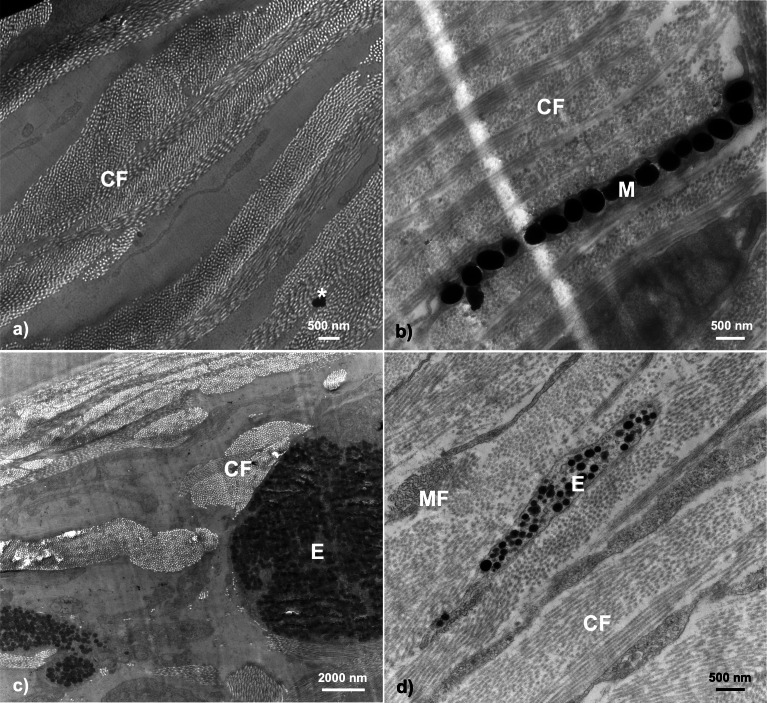
Examples of TEM micrographs of threespine stickleback throat dermal tissue. Analysis revealed collagen fibrils to be present in all samples, with melanosomes being present in samples taken from fish ranging in color from 1–5. Micrographs taken from fish across sexes, populations, morphologies, and ecotypes: (A) Little Campbell Anadromous female containing no dermal pigmentation; (B) Little Campbell Stream female containing a thin layer of melanosomes; (C) Little Campbell Anadromous male containing a large erythrophore with densely packed carotenoid vesicles; (D) Little Campbell Stream female containing an irregularly shaped erythrophore layer extending widely across skin but packed with many vesicles varying in electron density. M, melanophore; CF, collagen fibrils; MF, microfilaments; E, erythrophore; Asterisk (*), flaw in section (staining remnant). Scale bars range from 500 nm–2,000 nm as noted on each micrograph.

In general, transmission electron microscopy revealed throat dermal tissue to be dense in structural collagen fibrils and to contain at least one chromatophore structure if any coloration was present (*i.e.,* color score 1–5) (Fig. 3).

None of the samples in this study revealed complete chromatophore units containing erythrophores, iridophores, and melanophores with specific orientations. However, we did find both erythrophores and/or melanophores to be notable features in fish with darkened or orange-red coloration, though these structures were often mutually exclusive (*n* = 15 had either a melanophore or erythrophore; *n* = 3 had both). No iridophores or guanine crystals were detected in samples of throat dermal tissue. Further, we did not detect any structures restricted to only a specific sex, population, or ecotype, but did detect specific pattens of expression at the sex, population, and life history (*i.e.,* freshwater-resident or anadromous) levels (Table 2).

**Table 2:** Dermal chromatophore components and structures detected across fish. Results from this study revealed collagen fibrils to be the most abundant component found in dermal tissue micrographs. Further, TEM analysis revealed erythrophores and/or melanophores to be present across the majority of samples above color score 1, including across color morphs, sexes, populations, and ecotypes.

**Population**	**Sex**	**Color**	**Color score**	**Erythrophore (Yes/No)**	**Melanophore (Yes/No)**	**Iridophore (Yes/No)**	**Collagen fibrils (Yes/No)**
Little Campbell Anadromous	Female	Dull (colorless)	0	No	No	No	Yes
Little Campbell Anadromous	Female	Dull (colorless)	0	No	No	No	Yes
Salmon River	Female	Dull	1	No	No	No	Yes
Bonsall Creek	Female	Dull	1	No	Yes	No	Yes
Bonsall Creek	Male	Dull (melanic)	1	No	Yes	No	Yes
Bonsall Creek	Male	Dull (melanic)	1	No	Yes	No	Yes
Salmon River	Female	Orange-red	4	No	No	No	Yes
Little Campbell Stream	Female	Orange-red	3	Yes	No	No	Yes
Little Campbell Stream	Female	Orange-red	5	Yes	No	No	Yes
Salmon River	Female	Orange-red	2	Yes	No	No	Yes
Salmon River	Female	Orange-red	2	Yes	No	No	Yes
Little Campbell Stream	Male	Orange-red	5	Yes	No	No	Yes
Little Campbell Anadromous	Male	Orange-red	4	Yes	No	No	Yes
Little Campbell Anadromous	Male	Orange-red	4	Yes	No	No	Yes
Salmon River	Male	Orange-red	4	Yes	No	No	Yes
Salmon River	Male	Orange-red	3	Yes	No	No	Yes
Little Campbell Stream	Female	Orange-red	2	Yes	Yes	No	Yes
Little Campbell Stream	Male	Orange-red	5	Yes	Yes	No	Yes
Little Campbell Stream	Female	Orange-red	3	Yes	Yes	No	Yes

### Structure of the skin—collagen fibrils

**Figure 4:**
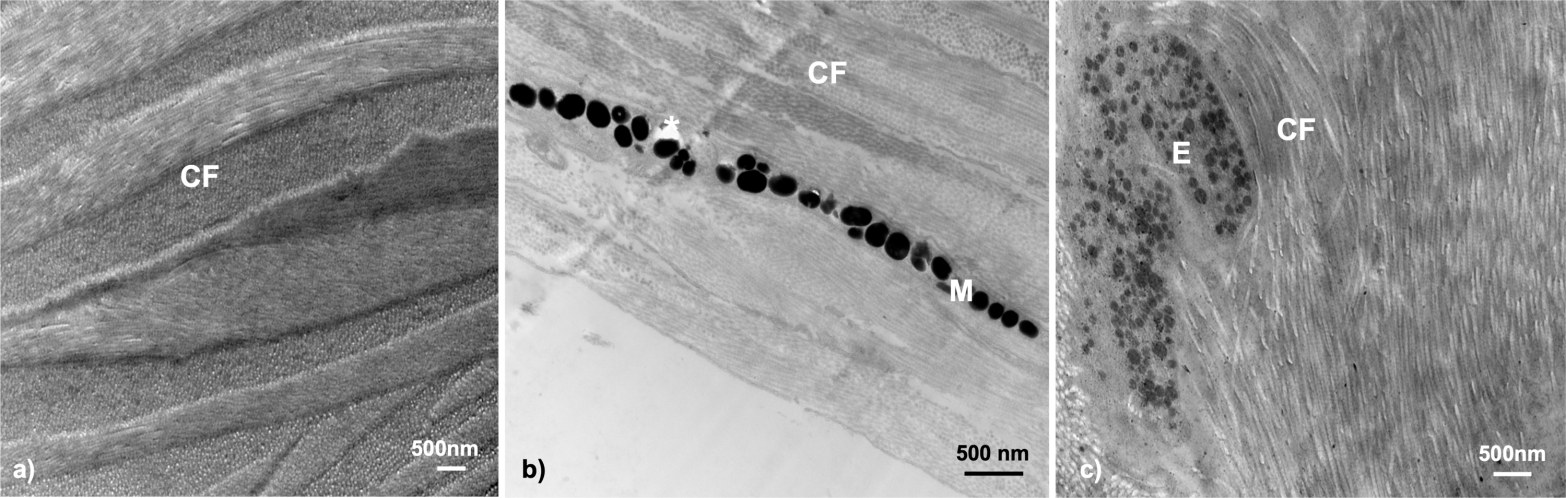
Throat dermal ultrastructure: collagen fibrils. Collagen fibrils were detected in all tissue samples. Collagen fibril networks are often closely interlaced with fibrils overlapping in characteristic patterns: (A) Little Campbell Anadromous female with densely packed collagen fibrils only; (B) Little Campbell Stream female with densely packed collagen fibrils within the skin and around a melanophore layer; (C) Salmon River female with long collagen fibril bundles enveloping the melanophore structure. CF, collagen fibrils; M, melanophore; E, Erythrophore; Asterisk (*), flaw in tissue section (hole). Scale bars all represent 500 nm length.

Transmission electron microscopy of the dermal throat tissue in *G. aculeatus* revealed commonality of tissue composition across fish. The throat tissue is very thin, yet sturdy in structure, owing to abundant collagen fibrils and extensive fibril networks (Fig. 4). Collagen fibrils were prominent features in all fish within this study (*n* = 19), and similar patterns were found across individuals of each sex, population, life history, and color expression type.

Collagen fibrils were the only structures found within the throat dermal tissue of one group of fish, Little Campbell Anadromous females (color score 0; *n* = 2), but appeared alongside chromatophore structures within the rest of samples where chromatophore components were detected (*n* = 15). These fibril networks often ran the length of the tissue analyzed and appeared alongside both larger and smaller chromatophore structures where such structures existed, namely erythrophores and melanophores. Further, collagen fibrils occurred not just in the spaces on either side of chromatophore structures, but appeared to interact with the chromatophore by wrapping around the component (Fig. 4C).

### Erythrophores

**Figure 5:**
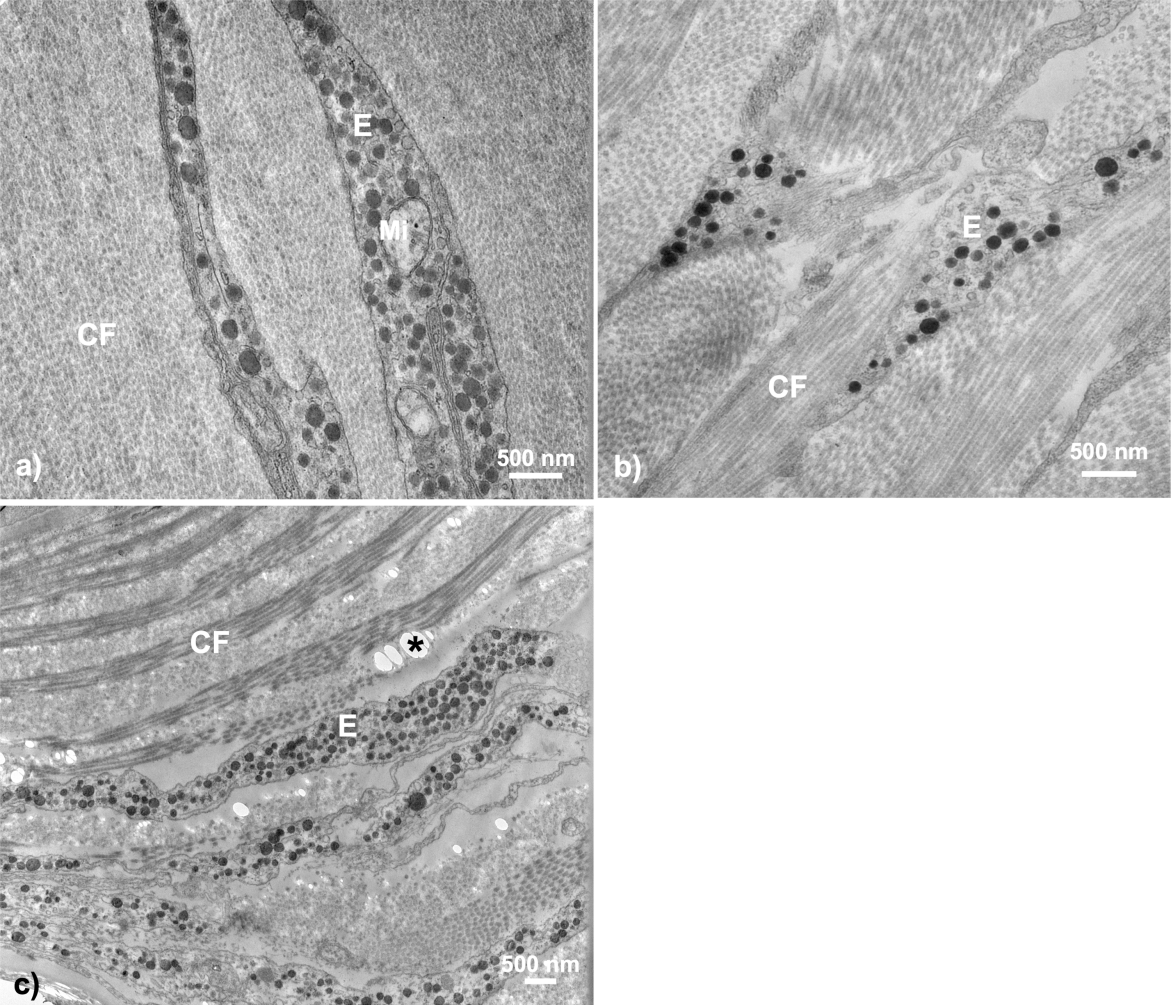
Throat chromatophore ultrastructure: erythrophores. The erythrophore was the most common dermal chromatophore structure found within throat tissue of fish with orange-red throat coloration. Erythrophores were largely characterized by cytoplasmic extensions containing circular structures ranging in color from grey to black in TEM images: (A) Salmon River male, (B) Little Campbell Stream female, (C) Little Campbell Stream male. E, erythrophore; CF, collagen fibrils; Mi, mitochondria; Asterisk (*), flaw in tissue section (hole). Scale bars represent 500 nm lengths.

The most evident and common pigmentary structure within the stickleback throat dermal tissue was the erythrophore (63.15% of all fish; *n* = 12). We did not find evidence of pterinosomes within any sample. Erythrophores were detected in almost all fish with orange-red color (visual score 2 to 5; *n* = 12 out of 13). The erythrophore units varied in morphology, with some appearing as cytoplasmic extensions with a thin layer of carotenoid vesicles, while others appeared bulkier in shape with irregularly interspaced vesicles within them (Fig. 3C). All erythrophores detected contained numerous darkened vesicles ranging from light grey to near black in color in unevenly distributed patterns (Fig. 5).

The presence of erythrophores was not confined to a single sex, population or ecotype. Further, Fisher’s exact test revealed a non-random association between erythrophore presence and orange-red coloration (two-tailed *P* = 0.003) across all fish. Dull fish were present across multiple populations, with both sexes from Bonsall and a single female from Salmon River expressing little coloration (color score 1), and only females within a single population, Little Campbell Anadromous, expressing no coloration (color score 0). In the one exception where an erythrophore was not detected in a fish above color score 1 (Salmon River colorful female; color score 4), no intact chromatophore components could be detected within the dermis. Lastly, only one population, Little Campbell Stream, with colorful males and both colorful and dull females, had individuals that contained both erythrophores and melanophores in some samples (*n* = 3 out of 6), though not in close proximity to each other.

### Melanophores

**Figure 6:**
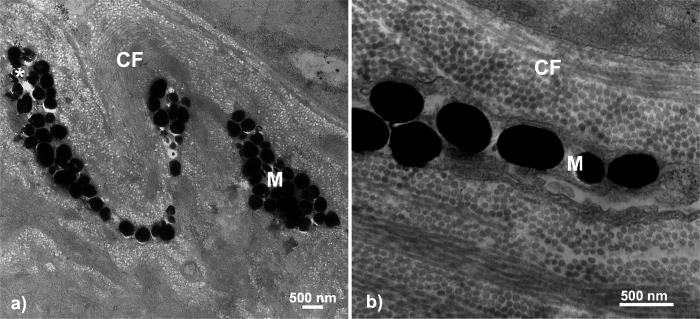
Throat chromatophore ultrastructure: melanophores. Evidence of melanophores appeared in a minority of fish and contained melanosomes with the classical black appearance ranging in size up to 500 nm: (A) Bonsall Creek female containing melanophores with numerous melanosomes; (B) Higher magnification view of a Little Campbell Stream female showing finer details of melanosome morphology. M, melanophore layer containing melanosomes; CF, collagen fibrils; Asterisk (*), flaw in tissue section (hole). Scale bars represent 500 nm length.

Melanophores were the only other chromatophore component found. In total, dermal melanophores were detected in 31.5% of the samples (*n* = 6 total; *n* = 3 male, *n* = 3 female) that ranged in color from 1 to 5 (*i.e.,* dull to colorful). Fisher’s exact test revealed no significant associations between melanophore presence and throat color characterization (*i.e.,* dull or orange-red; two-tailed *P* = 0.3201). The melanophore layers were detectable based upon the intracellular component, the electron-dense (black) melanosomes. The melanophore layers across fish were characterized by narrow, elongated cytoplasmic extensions that ranged in thickness from a single melanosome up to a few irregularly stacked melanosomes located in close proximity to each other. There was no evidence of multiple distinct melanophore layers being present within the same regions of skin. Melanosomes were largely dispersed across the tissue in broadly linear patterns and ranged in shape from circular to ovular with sizes up to 500 nm (Fig. 6).

Melanophores were detected within only some individuals of both sexes and color categorizations (*i.e.,* dull and colorful), indicating no broad color based phenotypic patterns. No melanophores were present in males or females inhabiting the single anadromous site, *i.e.,* Little Campbell Anadromous, nor from one of three freshwater sites, Salmon River. However, melanophores were present in both males and females inhabiting the other freshwater sites, including all fish from the Bonsall drainage (known to express more dull-darkened coloration) and one of each color expression type/sex from Little Campbell Stream. Fisher’s exact tests assessing non-random associations between melanophore expression and population comparisons between melanic (*i.e.,* Bonsall) and the more ancestor-like anadromous form (*i.e.,* Little Campbell Anadromous population) revealed significant associations (two-tailed *P* = 0.0286), with 100% presence in Bonsall fish and 100% absence in anadromous fish. Similarly, a test for associations of melanophore presence between populations with broad melanic expression, *i.e.,* Bonsall drainage, and all other populations again found significant, non-random associations of melanophore presence (Fisher’s exact test; two-tailed *P* = 0.0206).

## Discussion

We investigated the ultrastructure of throat dermal tissue in threespine stickleback that vary in the degree of orange-red throat nuptial coloration. We found collagen fibrils to be the most prominent component of dermal tissue, as well as some pigmentary structures, but no classical dermal chromatophore units. Of the chromatophore layers that were present, we found erythrophores to be the most widespread, followed by melanosomes. Erythrophores were common within the tissue of orange-red fish across sexes and populations, providing evidence of similar histological mechanisms in color production despite differentiation in genetic correlates between some populations (McKinnon, Newsome & Balakrishnan, 2022). We found population specific expression patterns in melanophores, with melanophores observed in 100% of Bonsall fish (males and females) and absent in 100% of Little Campbell Anadromous and Salmon River fish. We could not detect any iridophores, nor any evidence of chromatophore components in a group of fish with a 0 color score. Together these results suggest the erythrophore layer, likely containing carotenoid vesicles, to be the main component mediating variation in orange-red coloration. Melanosomes are important, as they are in many organisms, for darker coloration. Further, our findings suggest broadly similar chromatophore phenotypes across sexes and populations with similar color patterns. This is in contrast to inconsistent patterns in some gene expression results using fish from the same populations, but with an additional geographically distant set of fish included (McKinnon, Newsome & Balakrishnan, 2022).

### Dermal structure—collagen fibrils

Collagen fibrils are often a component of the dermis, which is a connective tissue layer that lies just under the epidermis but above any subcutaneous tissue (Lopez-Ojeda et al., 2021). This layer is fibrous in nature and functions to support and protect the skin and deeper structures from trauma, as well as to assist in temperature regulation, sensation, and housing of color specific cells in some animals. While the dermis contains many types of cells, collagen is one of the most important components within the extracellular matrix (including other connective tissues) and acts to provide the structural support needed to maintain integrity and strength of tissue (Shoulders & Raines, 2009). The threespine stickleback throat tissue is thin, non-scaled, located on the most ventral surface of the fish, and likely to be exposed to potential hazards, so would benefit from the structural support and integrity that dense collagen fibrils provide.

Collagen fibrils occur within the same spaces as chromatophore components, such as the erythrophore and melanophore layers containing the pigments driving coloration in the dermal tissue (*e.g.*, Bagnara, Taylor & Hadley, 1968; Fox & Whitear, 1990). Collagen has light scattering (*i.e.,* reflectivity) properties of its own and the biochemical/biophysical properties and arrangement of collagen fibrils within a tissue can also alter its reflectance (Brightman et al., 2000; Masuda et al., 2018). Together, these factors suggest an important influence of collagen density and location on the light reflective nature of skin. Additional research into how these collagen fibrils may influence coloration in stickleback throat dermal tissue is warranted as our results provide clear evidence of collagen fibrils within the dermal layer where chromatophore components reside.

### Dermal chromatophores and coloration

Melanophores are a common pigmentary structure across diverse taxa and darken the integument, where they are present, through melanin filled pigment granules called melanosomes (Bagnara, Taylor & Hadley, 1968; Bagnara & Hadley, 1973; Fujii, 1993). The color of integument-containing melanosomes can be influenced by both the density and dispersion of melanosomes within the melanophore, as well as the size and density of melanophores within the tissue. Many types of chromatophores have dendritic processes that extend in thin layers coincident with the plane of the skin (Fujii, 1993), including the melanophores we observed in *G. aculeatus*. Investigations in other animals, such as the electric ray (*T. ocellata*), have revealed blackened areas of the skin to contain many melanosomes that differ in size, but browner (*i.e.,* less dark) areas of the skin to be characterized by thin layers of smaller melanosomes sized less than 0.5 µm (see Taylor & Bagnara, 1972). While our fish do not present any obvious broad black/brown coloration at the macroscopic level, nor any large melanophore spots (though examples of minute darkened spots can be seen in Fig. 1), the appearance of melanosomes was similar to that described above, *i.e.,* thin layers of melanosomes varying in size up to 0.5 µm, which may explain the presence of some darkened coloration in Bonsall fish (score of 1 on the visual based scale).

Melanophores act to darken the integument by reducing the total amount of light reflected (Grether, Kolluru & Nersissian, 2004), but how dark the skin becomes depends upon the specific characteristics of the chromatophore and melanophore (Sköld, Aspengren & Wallin, 2002). Without the presence of many densely distributed melanophores, it is possible the dispersion of melanosomes would act to slightly darken the integument without creating macroscopically evident blackened spots or broad richly dark areas. Indeed, the patterns in melanophores detected here, *i.e.,* present within all dull Bonsall fish (color score 1), absent in all anadromous fish as well as one freshwater population, and in 50% of the fish within the remaining freshwater population, indicate the importance of melanophores in both sexes and at a population (as well as possibly life history) level. Fish sometimes appear to have slight darkened spots along the throat dermal tissue, and males of one specific population addressed here, *i.e.,* Bonsall fish (*n* = 3, color score =1), generally exhibit more darkened, melanic throat integument within field and laboratory settings (McKinnon, Newsome & Balakrishnan, 2022).

While melanophores often produce darkened tissue, xanthophores/erythrophores yield coloration ranging from yellow to orange-red. Pteridine-containing pterinosomes were absent in our micrographs, and pterins were not detected within throat tissue of fish from Salmon River in a preliminary assay, suggesting orange-red coloration in our study populations resulted largely from carotenoids within erythrophores. This is consistent with suggestions of carotenoid importance in *G. aculeatus* (Wedekind et al., 1998; Nordeide, Rudolfsen & Egeland, 2006, Pike et al., 2011).

While previous research in stickleback has found carotenoids to be the primary pigment involved in the production of orange-red coloration, with at least three different carotenoids being involved (see Wedekind et al., 1998), it is possible that additional pigments aid in the production and variation of red coloration. The presence of both erythrophores and melanophores in some of our throat samples also suggests a possible interaction between erythrophores and melanophores in orange-red color variation in stickleback. However, since the majority of cases where erythrophores were detected had only erythrophores and not melanophores (*n* = 9 of 12), any role for melanophores must be limited. Within the fish that contained both erythrophores and melanophores, possible interaction between the components could not be addressed or determined as the different chromatophores did not appear in close proximity to each other.

Alongside TEM ultrastructural analyses, additional methods such as quantification of chromatophores using light microscopy visualization of thick sections (similar to methods in MacDonald, 2020), and/or mass spectrometry and/or high performance liquid chromatography (HPLC) (*e.g.*, Wedekind et al., 1998), ideally from the same fish, could help to identify and quantify pigments involved in throat color variation. Further, assessment of the influence of collagen fibril density and distribution within dermal regions containing chromatophore components could help elucidate the role that such arrangements may play in the production and expression of color within stickleback throat tissue.

## Conclusion

Our work is the first to explore the cellular and histological structure of throat dermal tissue in threespine stickleback, as well as the ultrastructural composition of dermal orange-red coloration. We found collagen fibrils to be present in 100% of our samples, indicating they are an integral part of the dermal tissue composition and function in throat tissue. We found no fundamental chromatophore or pigmentary difference in orange-red color production across sexes or populations with similarly colored fish, as indicated by the widespread association of erythrophores with such color. Lastly, we found melanophore expression to be more population specific and associated with darkened tissue in both males and females.

## Additional Information and Declarations

### Field Study Permissions

The following information was supplied relating to field study approvals (i.e., approving body and any reference numbers): Field collections of threespine stickleback were approved by the British Columbia Ministry of Forests, Lands, and Natural Resource Operations (NASU14-92909, MRNA17-262956; MRSU18-290075) and the Semiahmoo First Nation.

### Data Availability

The following information was supplied regarding data availability: The raw data is available in Table 2.
